# Prophage-mediated endolysis of *Latilactobacillus Sakei* TMW 1.1290 induced by exogenous stress arising from food technologies

**DOI:** 10.1186/s12866-025-04161-7

**Published:** 2025-07-14

**Authors:** T Bardischewski, S Vallo, R Nitzsche, P Chanos, T Sieksmeyer, C Stühmeier-Niehe, K Aganovic, C Hertel

**Affiliations:** 1 DIL, German Institute of Food Technologies, Prof.-von-Klitzing-Str. 7, Quakenbrück, 49160 Germany; 2 Institute of Food Quality and Food Safety, University of Veterinary Medicine Hannover, Foundation, Bischofsholer Damm 15, 30173 Hannover, Germany; 3 Elea Technology GmbH, Professor-von Klitzing Str. 9, Quakenbrück, 49610 Germany; 4 DIL Technologie GmbH, Prof.-von-Klitzing-Str. 7, Quakenbrück, 49160 Germany

**Keywords:** Prophage induction, Starter cultures, Lytic enzymes, HHP, UV, PEF, RT-qPCR

## Abstract

**Background:**

Intracellular enzymes released during bacterial cell lysis contribute to the development of flavor and texture in fermented foods through the hydrolysis of proteins and fats. In fermented meat products this takes place only late in the fermentation process which limits its effect. Therefore, time-controlled cell lysis constitutes a potentially valuable tool for enhancing sensory characteristics of the final product. This study investigated the prophage-induced, time-delayed bacterial cell lysis in *L. sakei* TMW 1.1290 by using different stress conditions, including those generated by food technologies, like pulsed electric fields (PEF) and high hydrostatic pressure (HHP).

**Results:**

UV light induced transient cell lysis, confirmed by a 2102-fold increase of expression of the prophage amidase gene, as determined by Reverse Transcription quantitative Polymerase Chain Reaction (RT-qPCR). The effect of oxidative stress chemicals like menadione and hydrogen peroxide was far more pronounced, causing evident cell lysis, although probably differing in their modes of action. Although PEF treatment and HHP are known to cause direct or indirect oxidative stress in cells, their applications on cell suspensions at sublethal conditions were not able to cause cell lysis induction under the experimental conditions used.

**Conclusions:**

Prophage-mediated cell lysis in *L. sakei* TMW 1.1290 can be induced by UV light and mitomycin C, while H₂O₂ and menadione showed prophage activation potential but significantly inhibited growth. Food technologies like PEF and HHP were ineffective at inducing prophage-mediated cell lysis despite causing sublethal injury, requiring further study of treatment parameters.

## Background

The usage of starter cultures in raw fermented sausages does not only contribute to the safety of the final product, but also to development of its organoleptic characteristics. Commonly, meat starter cultures consist of coagulase-negative staphylococci (CNS) and lactic acid bacteria (LAB) consortia [1]. While CNS contribute to the final color and taste of fermented sausages, lactic acid bacteria primarily convert naturally occurring and added sugars in the meat batter to lactic acid [2]. However, LAB also contribute to the hydrolyzation of meat proteins and fat through intracellular enzymes, released during cell lysis [3], resulting in the formation of aroma precursor compounds. Subsequent chemical reactions and microbial metabolism convert these precursors to fermented meat volatile aroma compounds, like aldehydes, contributing to the sensory characteristics of the final product [4, 5]. Indeed, intracellular enzymes of LAB, either released due to autolysis or added in purified form in the fermentation, accelerated ripening and development of flavor in fermented meat but also milk products [6–9].

The bacterial cell lysis can be triggered by various factors during fermentation or through pre-treatment of cells [10–12]. The process occurs through the activation of cell wall hydrolases (CWH) which, depending on their specific catalytic activity, origin and function, can be categorized as autolysins, exolysins and endolysins [13, 14]. The endolysins in particular are encoded by genes within prophages – segments of bacteriophage DNA that have integrated into the bacterial chromosome during lysogenic infection. These prophages remain dormant within the host cell until activated, at which point they produce endolysins that degrade the cell wall of their host cell to release newly formed phage particles [15]. The induction of the prophage represents the switch from the dormant lysogenic to the active lytic replication cycle, typically activated by the SOS response due to DNA damages [16]. Common inducers of the SOS-response include UV light and mitomycin C [17, 18].

Apart from those, other stress-response inducing parameters or techniques, particularly suited for application in food production, can similarly activate the SOS response and induce prophage mediated endolysin production. These include changes in temperature and pH, as well as, osmotic pressure or low nutrient concentrations, with promising results *in vitro* [19, 20]. Other examples include high pressure [21, 22] or the extracellular and/or intracellular Radical Oxygen Species (ROS) generation [20, 23, 24]. To this end, high hydrostatic pressure (HHP) – a non-thermal food pasteurization method involving application of pressures up to 600 MPa for a short time– and pulsed electric fields (PEF) – a non-thermal technique applying brief high-voltage electrical pulses– can be considered as food grade methods for the induction of prophages in cultures [25–28]. Through the induction of early cell-lysis of the starter culture in fermented sausage production, an increase in the concentration of volatile aroma compounds and therefore, an improvement of the sensory characteristics of fermented sausages may be achieved. HHP and PEF treatment equipment are industrially available at different modes (continuous/batch), sizes and/or throughputs and may be easily used as adjunct processes to already established lines of fermented sausage production for the pre-treatment of starter cultures before their incorporation in the food matrix. All the above can be of high importance especially to small and medium size producers which, through small adjustments to their production lines, may be able to create signature products with unique sensory characteristics.

The goal of this study was to evaluate HHP and PEF as inducers of a time-delayed prophage mediated endolysis in a *Latilactobacillus sakei* strain bearing prophage-encoding DNA and to compare with known triggers like UV light, mitomycin C and hydrogen peroxide. This was attempted in view of the potential benefits of utilization of such techniques, particularly PEF and HHP, in prophage induction and programmed cell-lysis in industrial lines of fermented sausage production.

## Methods

### Microorganisms

The two strains of *L. sakei* TMW 1.1290 and TMW 1.2292 utilized in this study were provided by the Department of Microbiology, Technical University of Munich (TUM). Strain *L. sakei* TMW 1.1290 (hereafter prophage-harbouring), was originally isolated from fermented sausage and contained one intact prophage (P1), which is inducible by mitomycin C or UV light as previously confirmed using transmission electron microscopy [17]. In contrast, the second strain *L. sakei* TMW 1.2292 (hereafter prophage-free) was a derivate of strain TMW 1.1398 [29]. The strains were maintained at −20°C and − 80°C with 25% (v/v) glycerol, until usage.

### Preparation of precultures

Overnight cultures were prepared in 15 ml sterile centrifuge tubes, by adding 15 µl of the frozen stock suspension in 15 ml fresh modified De Man, Rogosa and Sharpe (mMRS) broth [17], with sterile glucose added after autoclaving. The cultures were incubated at 30°C overnight static to obtain a preculture. Optical density at 600 nm (OD_600_) was measured in a 48 well plate (Greiner Cellstar, Merck KGaA,) in a volume of 1 ml per well (optical pathlength = 1 cm, top to bottom; 25 flashes; bandwidth: 9 nm, Infinite Pro 200, Tecan) and the OD_600_ of sterile mMRS broth was subtracted.

### Prophage induction assay

The ability of different treatments to induce prophages and cause cell lysis of prophage-harbouring strain TMW 1.1290 was evaluated by monitoring the growth kinetics of the culture after it was submitted to any specific treatment and comparing them to the kinetics of prophage-free strain TMW 1.2292. Increase of OD_600_ indicated growth while decrease in OD_600_ indicated cell lysis. Treatment with mitomycin C from *Streptomyces caespitosus* (Sigma-Aldrich, St. Louis, MO, USA), added just before the start of the kinetics served as positive control for prophage induction and cell lysis. Negative controls were cultures receiving either no physical treatment or for which no inducing chemical was added. Precultures, prepared as described earlier, were diluted with fresh mMRS medium to an OD_600_ of 0.05 and incubated at 30°C until it entered the early logarithmic phase (2 h), obtaining working cultures. This time point in the logarithmic phase of the preculture was selected based on preliminary experiments, in which the incubation time of the preculture was varied from 1 to 5 h in order to be able to showcase a clear drop in the OD_600_ of the working culture after treatment with mitomycin C in a concentration range of 0.5 to 20 µg/ml. All treatments (chemical or physical) were done in working cultures. The evaluation of growth kinetics of treated and control cultures were done by measuring the OD_600_ of the cultures during incubation at 30°C for at least 12 h and in technical duplicates. OD_600_ measurements were done in 48-well plates as before, every five minutes following shaking (orbital, 3.5 mm radius, 5 s) using the i-control software (v. 1.12, Tecan).

### Exposure to UV light

UV light treatment of working cultures was done in a gel documentation UV–light bed (AlphaManager, Alpha Innotech, San Leandro, CA, USA) at 302 nm for 8 min. Fifty milliliters of the working culture were treated in baffled, 500 ml Erlenmeyer flasks. The flasks were placed directly on top of four UV fluorescent lamps situated under the glass slab of the UV–light bed. The power per area at the top of the glass slab was measured at about 25 W/m^2^ (Field Mate Laser Power Meter, Coherent Inc.). For an 8-minute treatment this corresponded to a light energy of 12 kJ/m^2^ or 114 J per sample calculated from the diameter of the round area that the liquid covered at the bottom of the Erlenmeyer flask (about 11 cm). Following treatment, one milliliter from each flask was transferred to a well of a 48-well plate and assayed for prophage induction as described before. Furthermore, the rest of the culture in flasks was incubated static at 30°C and a volume of one milliliter was collected after 0, 1, 2 and 3 h of incubation. After centrifugation the resulting pellets were frozen at −20°C for subsequent RT-qPCR analyses. For positive and negative controls, flasks with fifty milliliters of working culture each were kept at room temperature. For positive controls, mitomycin C was added before the beginning of the prophage induction assay.

### Exposure to hydrogen peroxide and menadione

For hydrogen peroxide and menadione treatments, 10 µl of a 200 mM hydrogen peroxide (H_2_O_2_, 30%, Carl Roth) stock solution or 5 µl of a 100 mM stock solution of menadione (vitamin K3, Sigma-Aldrich) in dimethyl sulfoxide (DMSO) were added to 1 ml of the working culture just before the phage induction assay. This way, concentrations of 2 mM and 0.5 mM were reached, respectively, in order to induce oxidative stress. Positive controls without DMSO were included as described earlier as preliminary experiments showed that the addition of DMSO did not exert any influence on the kinetics of the strains under investigation.

### Exposure to PEF

For PEF treatment, working cultures were diluted 1:1 with cold sterile water to adjust the cell suspension’s conductivity to around 7.5 mS/cm in order to prevent electric arcing. The treatments were done in an Elea PEFPilot™ Dual system (Elea Technology GmbH), using a 15 ml chamber with an electrode distance of 2 cm, in technical duplicates. The field strength was varied at two levels (3.75 kV/cm and 5 kV/cm) and the number of pulses were set to 61 and 35 pulses, respectively, to achieve an approximate specific energy level of 115 kJ/kg (Table 2) according to Eq. 1 where C is the capacity of the device in (1 µF). The pulse width and the frequency were kept at 8 µs and of 21 Hz, respectively. The effect of the different PEF treatments on the cell counts of the cultures was evaluated by spread plating on MRS agar (Roth).

1 $$\:\frac{0.5\:\text{C}\:\times\:{\text{U}}^{2}}{\text{k}\text{g}\:\times\:1000}\times\:{\text{n}}_{\text{p}\text{u}\text{l}\text{s}\text{e}\text{s}}$$

After PEF treatment, the 15 ml of treated suspension were centrifuged (4000 *g*, 5 min, 4°C) and the cell-pellet was resuspended in 7.5 ml fresh mMRS reaching an OD_600_ of about 0.1. All samples were kept and transferred on ice for the phage induction assay in order to arrest growth and the development of any premature SOS response of the cells. One milliliter of the adjusted suspensions for each technical duplicate was transferred to a 48-well plate for the phage induction assay. Separate samples for positive and negative controls were handled in the same way (including dilution with water, centrifugation and resuspension in mMRS) with the difference that they were kept on ice at all times until being transferred to a 48-well plate. Induction of the positive control with mitomycin C was done as before.

### Exposure to HHP

For HHP 30 ml of working cultures were transferred in duplicate PET vials (Nipak HV, MD Lopik, Netherlands) closed tightly, sealed in separate plastic bags filled with water and treated at 200 MPa in a Hyperbaric 6000/55 batch plant for different pressurization times without temperature control. The vials with the treated samples were then kept on ice until transferred for the phage induction assay. Negative and positive control samples were prepared and handled in the same way, including storing and transferring them on ice, but not treated with HHP. Induction of the positive control was with mitomycin C was done as before. The pressure and time range were selected based on preliminary experiments as the most severe treatment conditions causing no significant (*p* > 0.05) cell count reduction (Table 3).

### Primer design

To determine expression of gene *ampD* coding for the N-acetylmuramoyl-L-alanine amidase-like protein, three housekeeping genes (*recA*,* gyrB* and *dnaG*) were used for comparison. Primers with a length of about 20 bp and a melting temperature (T_m_) of 60°C for an amplicon length up to approximately 150 bp were derived from the corresponding gene sequences of prophage-harbouring strain TMW 1.1290 (accession number: JAHIAJ000000000) using software ApE v3.1.3 (David & Jorgensen [30]). The suggested primers were inserted into Serial Cloner v2.6.1 [31] to verify the possible PCR amplicon in silico. Each primer was checked on its free energy through RNAfold WebServer [32] to choose only primer without any secondary structure and a free energy higher than − 4 kcal/mol. The primer pairs used are listed in Table 1.

**Table 1:** Designed primer for expression analysis of mRNA samples of *L. sakei* prophage-harbouring strain TMW 1.1290 for RT-qPCR

Primer name	Sequence 5’ – 3’	T_m_ [°C]	Amplicon length [bp]	dimer dG [kcal/mol]	T_m_ primer pair [°C]
recA forward	CTGCTCTAACCTTCAAACGA	58.0	111	−0.09	60.3
recA reverse	TCTTGGTACTCTTATGGCTCAG	59.4			
DNAgyr forward	AGAAACAGAAGCCCAAATTGAG	59.6	153	−0.08	58.4
DNAgyr reverse	CCAAACCAAATGATGTAACCCT	59.3			
DNAprimase forward	AAGCAACTCTATCAACAAGCCA	60.7	116	−0.27	63.5
DNAprimase reverse	CTCAATTAACGCATCGGTCATCTG	62.5			
Amidase forward	TTATCCATCACAACGCAACAACC	62.4	122	−0.35	63.5
Amidase reverse	CTTCAACGCATCCAATAATCTCGG	62.6			

### RNA isolation and cDNA synthesis

RT-qPCR was used to assess the gene expression of the chosen prophage lytic enzyme -acetylmuramoyl-L-alanine amidase (EC 3.5.1.28). A modified procedure was used to extract RNA from fresh samples, which involved centrifuging the recovered cell pellet once again and resuspending it in RNA Protect (Qiagen, Venlo, Netherlands). The protected cells were stored at −20°C for a maximum of one week until the RNA was further purified. Using 200 µl of lysis buffer (30 min, 37°C) containing 20 units of mutanolysine from *Streptomyces globisporus* ATCC 2155 (Sigma-Aldrich) and 20 mg/ml of lysozyme (Serva Electrophresis GmbH, Heidelberg, Germany) in 30 mM Tris and 10 mM EDTA at pH 6.2, RNA release was achieved by enzymatic digestion following the protocol from Villa-Rodríguez et al. [33]. Following a five-minute incubation period, 1000 µl of TriZol (Invitrogen Corp.) and 200 µl of chloroform (Merck KGaA, Darmstadt, Germany) were added to the suspension and mixed by inverting. RNA was extracted from the clear aqueous supernatant and purified using the Monarch total RNA Kit (New England Biolabs (NEB)) before being eluted in 30 µl deionized water following a 15-minute centrifugation at 12,000 *g* and at 4°C. To perform DNA digestion, 10 µl of DNAse I and 10 µl of DNAse buffer were added, and the mixture was incubated for an hour at 37°C. Lastly, the Monarch Total RNA Kit was used to purify the RNA once more. The purity, concentration and integrity of the RNA preparations were confirmed with the Nanodrop (Thermo Fisher Scientific) the Qubit™ (Thermo Fisher Scientific) and the Tapestation (Agilent Technologies, Inc., Santa Clara, CA, USA) devices, respectively.

Using the Luna Superscript Kit (NEB), cDNA synthesis was carried out in 20 µl mixtures according to manufacturer’s guidelines and processed in a thermocycler (BioRad) as follows: 2 min at 25°C for primer annealing, 10 min at 55°C for cDNA synthesis, and 1 min at 95°C for heat inactivation of the enzyme. Negative controls for testing the presence of residual DNA in the RNA samples was prepared using the same RT Supermix without reverse transcriptase. The cDNA was either used right away or kept frozen at −20°C until use in the qPCR assay.

### Reverse transcription quantitative polymerase chain reaction (RT-qPCR)

The cDNA was used in 10 µl qPCR reactions in 96-well BioRad plates employing a master cycler, operated by the BioRad CFX Maestro software 1.1 (v. 4.12433.1219). One µl of cDNA sample and 5 µl of BioRad’s SsoAdvanced Universal SYBR GREEN Supermix were used for the reaction. Following the addition of primer pairs at a final concentration of 200 µM for each primer, 10 µl of PCR-grade water was added. After vortexing and brief centrifugation the plate was sealed with BioRad plastic foil. The master cycler was set up as per protocol, starting with the first denaturation step at 95°C for 3 min. This was followed by 39 repetitions of the cycle steps of denaturation and annealing at 95°C for 15 s and 55°C for 30 s, respectively. Following qPCR, a melting curve analysis was run, raising the temperature by 0.5°C every 5 s, from 65°C to 95°C to assess whether single specific products were synthesized.

### Calculation of the relative gene expression ratio

Following qPCR, the analysis for the relative gene expression was performed by using the cycle step for each sample where the fluorescence value rose above a predetermined threshold (Ct). The difference between the Ct values of the treated and untreated sample groups provided the ΔCt values. The normalized relative quantity (NRQ, Eq. 2) of the gene of interest (goi, Eq. 2) in relation to the selected housekeeping genes (ref, Eq. 2) was calculated for the purpose of analyzing the various expression ratios between different samples. This was done with correction of the determined specific primer efficiency (E, Eq. 2), as follows [34, 35]:

2 $$\:\text{N}\text{R}\text{Q}=\frac{{\text{E}}_{\text{g}\text{o}\text{i}}^{\varDelta\:\text{C}\text{t},\text{g}\text{o}\text{i}}}{\sqrt[\text{f}]{\prod\:_{\text{o}}^{\text{f}}{\text{E}}_{{\text{r}\text{e}\text{f}}_{\text{o}}}^{\varDelta\:\text{C}\text{t},{\text{r}\text{e}\text{f}}_{\text{o}}}}}$$

where “f”: the number of reference genes.

The efficiencies of each pair of primers were calculated by a linear regression of used log dilutions of cDNA and the estimated Ct values. The defined formula Eq. 3 was used to calculate the reaction efficiency:

3 $$\:\text{E}={1+10}^{\frac{-1}{\text{s}\text{l}\text{o}\text{p}\text{e}}}$$

### Statistical analysis

Statistically significant differences in the surviving counts of PEF or HHP treated cultures were evaluated on the average survivals of 3 replicate samples t-test and compared to the respective average survival of the control (not treated) using t-test in MS Excel at a significance level of 95%.

## Results

### Treatment with UV light

**Fig. 1:**
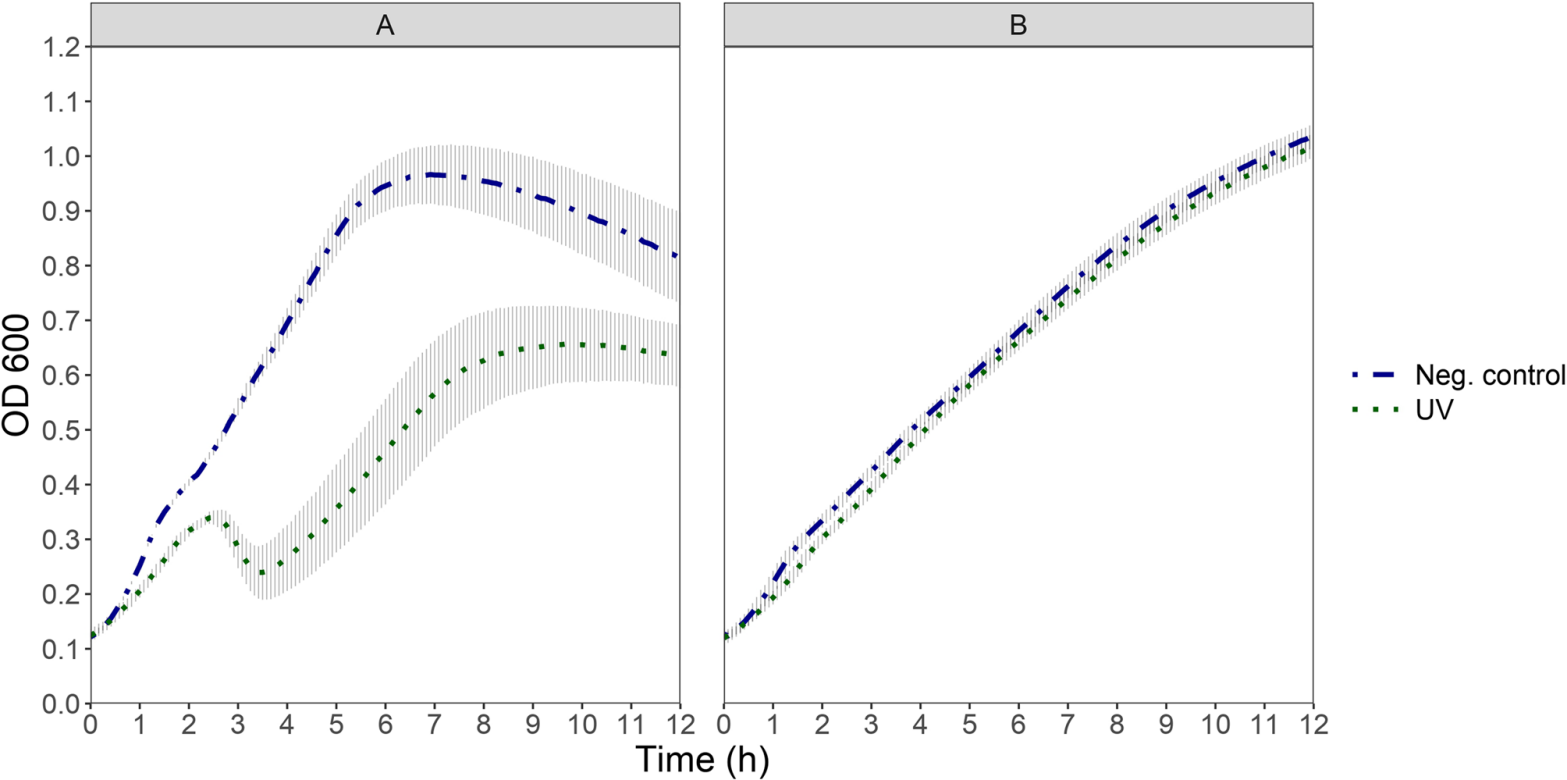
OD_600_ development of UV treated cultures of prophage-harbouring strainTMW 1.1290 **A** and prophage-free strain TMW 1.2292 **B** in a plate reader at 30°C. The treatment was for 8 min with UV light at a wavelength of 302 nm compared to the respective not treated samples from each strain (Neg. control). Each point shows the average of three independent experiments (biological replicates), error bars indicate ± 1 standard deviation. The treatment took place at t = 0 h

**Fig. 2:**
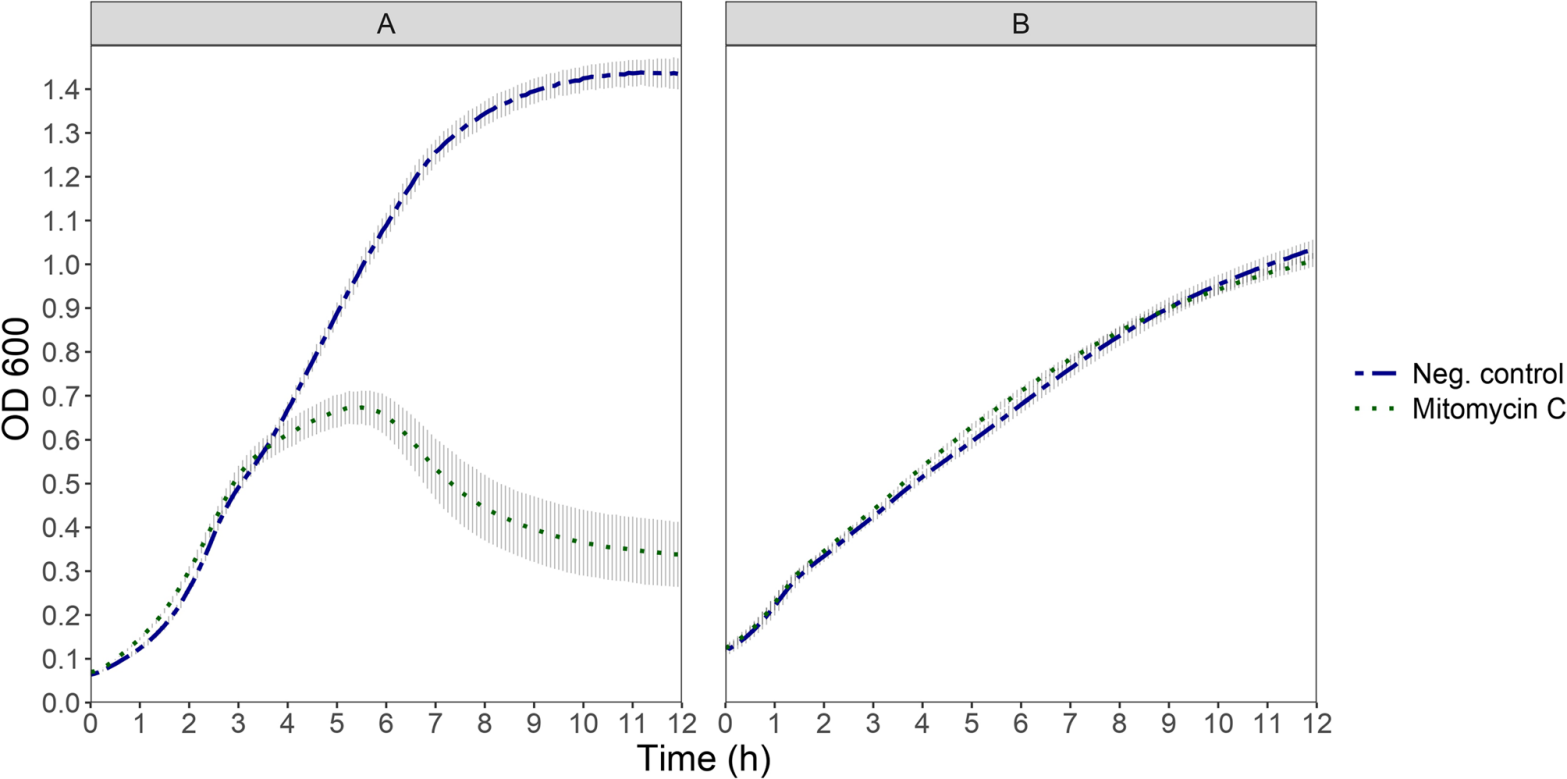
OD_600_ development of cultures of *L. sakei* prophage-harbouring strainTMW 1.1290 **A** and prophage-free strain TMW 1.2292 **B** at 30°C after addition of mitomycin C (20 µg/ml) compared to their not treated samples (Neg. control). Each point shows the average of three independent experiments (biological replicates), error bars indicate ± 1 standard deviation. The treatment took place at t = 0 h

Following UV light treatment, the OD_600_ of the culture of prophage-harboring strain TMW 1.1290 increased steadily and peaked at around 2.5 h after the treatment (Fig. 1). Compared to the OD_600_ development of the untreated control, the OD_600_ decline of the treated culture after 2.5 h represents the lysis induced by the treatment. Following an approx. 1-hour decline, the OD_600_ rose again for a second growth phase which lasted from that timepoint until it entered the stationary phase, roughly 6 h after the treatment. In the positive induction control with mitomycin C (Fig. 2) the decrease of the measured OD_600_ in time was greater than that caused by the UV light treatment. Following the mitomycin C induced lysis, the cells did not recover, as evidenced by the continuous decrease in OD_600_. As expected, no lysis response in the cells of the prophage-free strain TMW 1.2292 was observed after treatment of the culture with either UV light or mitomycin C (Figs. 1 and 2).

### Exposure to ROS

The experiments with hydrogen peroxide and the ROS-generating chemical compound menadione were used to investigate the inducibility of prophage lysis by oxidative stress in prophage-harbouring strain TMW 1.1290 as compared to the prophage-free strain TMW 1.2292.

**Fig. 3:**
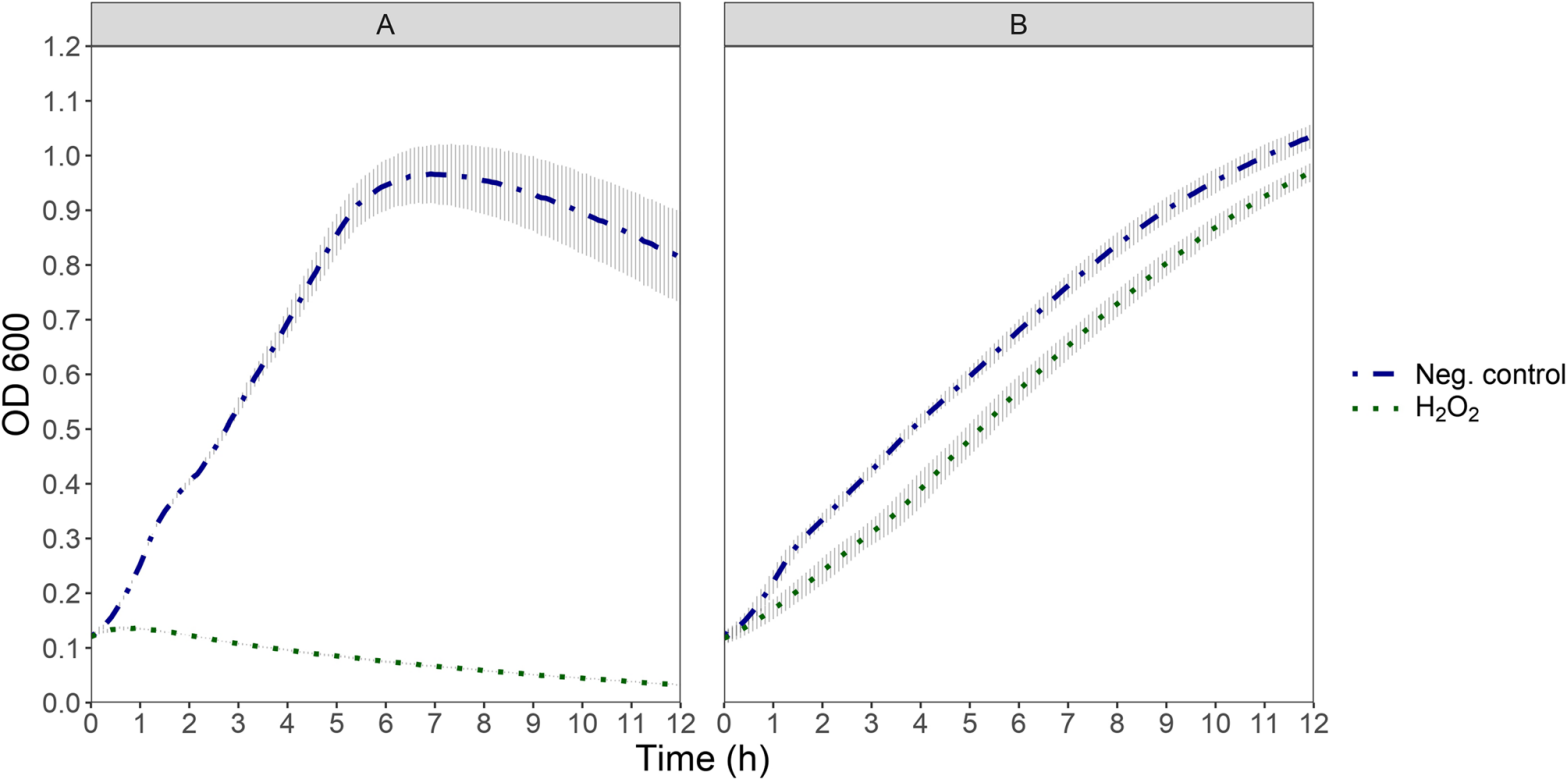
OD_600_ development of cultures of prophage-harbouring strain TMW 1.1290 **A** and prophage-free strain TMW 1.2292 **B** at 30°C after addition of 2 mM hydrogen peroxide compared to their not treated samples (Neg. control). Each point shows the average of three independent experiments (biological replicates) error bars indicate ± 1 standard deviation. The treatment took place at t = 0 h

**Fig. 4:**
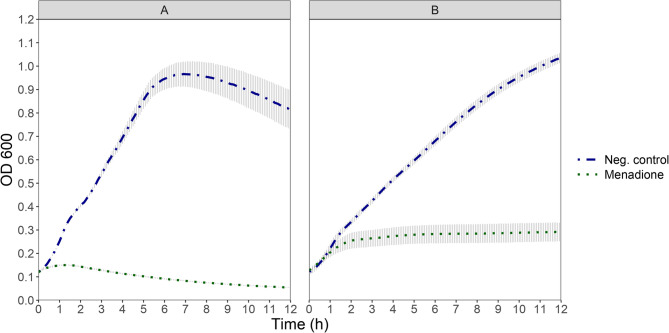
OD_600_ development of cultures of prophage-harbouring strain TMW 1.1290 **A** and prophage-free strain TMW 1.2292 **B** at 30°C after addition of 0.5 mM menadione compared to their not treated samples (Neg. control). Each point shows the average of three independent experiments (biological replicates), error bars indicate ± 1 standard deviation. The treatment took place at t = 0 h

In general, the treatment of both prophage-harboring and prophage-free cultures with hydrogen peroxide or menadione resulted in lower OD_600_ values throughout the incubation time when compared each to their untreated controls, indicating growth inhibition (Figs. 3 and 4). OD_600_ of the prophage-harbouring strain TMW 1.1290 main culture exhibited a slight increase over an OD_600_ value of just 0.1 at around 0.5 h after the addition of hydrogen peroxide and was reduced thereafter to almost 0 by the end of the measurement period (Fig. 3A). The effect of the addition of menadione was similar with the difference that the peak OD_600_ occurred just under two hours after the addition of the chemical (Fig. 4A). In contrast, prophage-free strain TMW 1.2292 exhibited a transient reduced growth rate after addition of hydrogen peroxide, compared to its untreated control. However, growth was not completely inhibited, as the OD_600_ continued to increase until the end of measurement (Fig. 3B). Interestingly, addition of menadione to the prophage-free strain TMW 1.2292 culture led to initial growth, reaching stagnation with OD_600_ of around 0.25 until the end of experiment. This decrease of the culture OD_600_ of prophage-harbouring strain TMW 1.1290 after addition of either H_2_O_2_ or menadione, as compared to partial or full growth of culture of prophage-free strain TMW 1.2292 indicated that oxidative stress through ROS generation might induce the intact prophage and the cell lysis. This finding prompted us to test PEF as an oxidative stress inducing method, as the technology is known to generate intracellular and extracellular ROS [25].

### Exposure to PEF and HHP

The intensity of PEF treatment was chosen to be at the threshold between sublethal cell injury and inactivation for the strains. Earlier experiments showed that the field strength could not be increased further than 5 kV/cm in diluted mMRS with conductivity of 7.5 mS/cm due to the occurrence of electric arcing around the treatment chamber. The PEF conditions of the treatments applied and their effect on the cell counts of prophage harbouring-strain TMW 1.1290 and prophage-free strain TMW 1.2292 are displayed in Table 2.

**Table 2:** Inactivation of the two investigated strains treated with PEF. Mean CFU counts (*n* = 3)

Sample	Field strength (kV/cm)	Number of pulses	Pulse width (µsec)	Frequency (Hz)	Specific energy (kJ/kg)	Surviving counts Log_10_ (CFU/ml) ± SD	
Strain						TMW 1.1290	TMW 1.2292
PEF1	3.75	61	8	21	114,4	7.71 ± 0.05	7.81 ± 0.12
PEF2	5	35	8	21	116,7	7.5 ± 0.06*	7.75 ± 0.11
Control	/	/	/	/	/	7.82 ± 0.06	7.82 ± 0.1

*SD* Standard deviation

*significant difference compared to the control (ttest, *p* < 0.05)

**Fig. 5:**
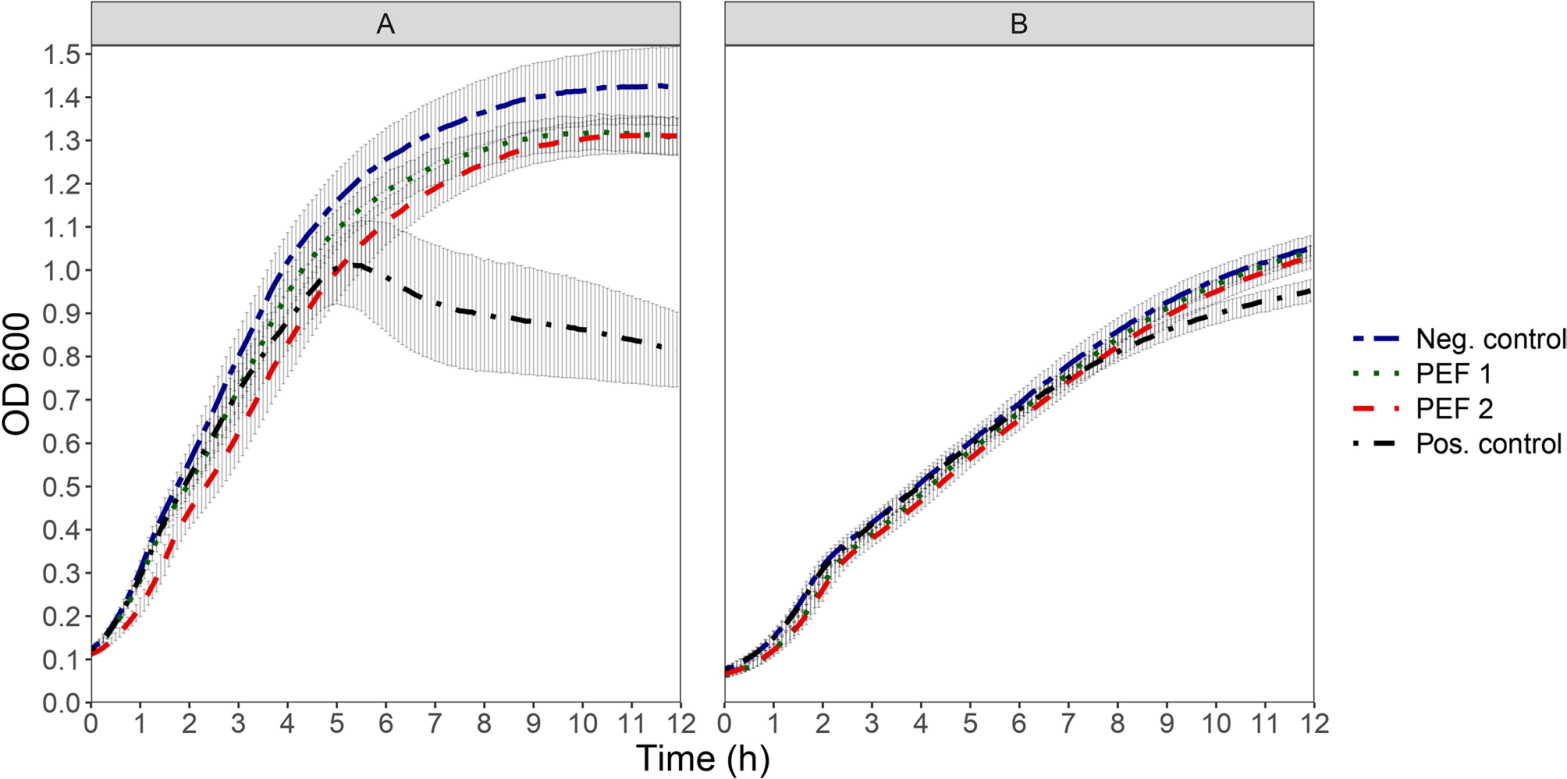
OD_600_ development of prophage harbouring-strain TMW 1.1290 **A** and prophage-free strain TMW 1.2292 **B** after treatment with PEF. Samples studied were either not treated (Neg. control), treated with 5 µg/ml mitomycin C (Pos. control), treated for 61 pulses at 3.75 kV/cm (PEF1), or treated for 35 pulses at 5 kV/cm (PEF2). Each point shows the average of three independent experiments (biological replicates) and error bars indicate ± 1 standard deviation

The application of PEF2 treatment at a specific energy of 116.7 kJ/kg caused a significant (t-test, *p* < 0.05) reduction of 0.3 log_10_ CFU/ml in the cell counts of strain TWM 1.1290 compared to the control (Table 2). None of the PEF treatments had an effect on the cell counts of strain TMW 1.2292. The PEF treatment reduced both, the OD_600_ increase rate and the final OD_600_ of prophage-harbouring strain TMW 1.1290 at 12 h of incubation. After 12 h of incubation the OD_600_ of both PEF1 and PEF2 treated cultures were not significantly lower compared to that of the control culture (t-test, *p* > 0.05). The respective effect of PEF treatments on strain TMW1.2292 was negligible (Fig. 5). None of the treatments induced lysis on prophage-harbouring strain TMW 1.1290 as compared to the culture of the same strain treated with 5 µg/ml mitomycin C, in which lysis was evident.

Initial experiments with HHP showed that a treatment with 300 MPa for even one minute of holding time resulted in an inactivation of more than 3 log_10_ cfu/ml for both strains (data not shown). Subsequent experiments in the range of 100 to 250 MPa at varying holding times showed at 200 MPa a varying effect from sublethal injury to significant cell count reductions for prophage harbouring-strain TMW 1.1290. The pressure/holding time combinations which were used and their effect on the cell counts of both strains are displayed in Table 3.

**Table 3:** Inactivation of *L. sakei* strains by HHP (200 MPa). Mean CFU counts (*n* = 3)

Sample	Holding time (min)	Surviving counts log_10_ (CFU/ml) ± SD	
		TMW 1.1290	TMW 1.2292
1	1	7.54 ± 0.06	7.9 ± 0.07
2	2	7.48 ± 0.11	7.92 ± 0.09
3	4	7.48 ± 0.03*	7.82 ± 0.11
4	7	7.39 ± 0.06*	7.87 ± 0.06
Control	/	7.66 ± 0.09	8.02 ± 0.09
Mitomycin C Control	/	7.82 ± 0.14	8.06 ± 0.1

*SD* Standard deviation, / no HHP treatment

*significant different compared to the control (t-test, *p* < 0.05)

**Fig. 6:**
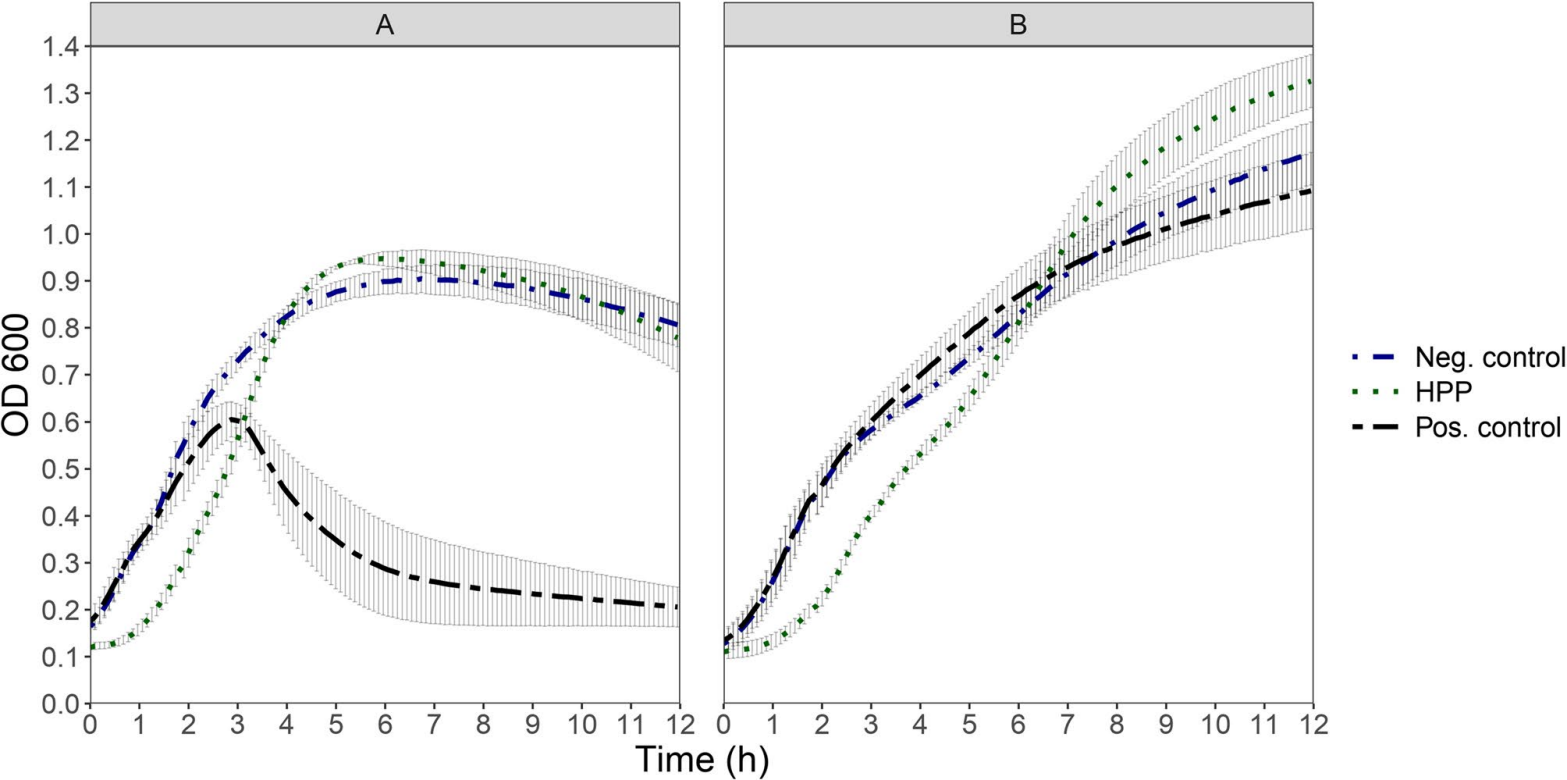
OD_600_ development of cultures of prophage harbouring-strain TMW 1.1290 **A** and prophage-free strain TMW 1.2292 **B** treated with HHP at 200 MPa for 7 min (HHP) or 5 µg/ml mitomycin C (Pos. control) or remained untreated (Neg. control). Each point shows the average of three independent experiments (biological replicates), error bars indicate ± 1 standard deviation. Other pressure holdings times below 7 min were not shown for clarity

The application of pressure at 200 MPa caused an increase in the lag phase of both cultures compared to untreated control. However, despite the time-delayed start of growth, the HHP treated cultures exhibited a higher growth rate and surpassed their controls in terms of OD_600_ at around 4 to 6 h of incubation. At the selected pressure (200 MPa), a reduction of the cell counts of the cultures directly after treatment was observed. This decrease was positively correlated with increasing holding time and became significant after 4 min of holding time compared to the untreated control (t-test, *p* < 0.05). At the end of the incubation, both control and treated cultures showed a slight decrease in OD_600_, possibly due to autolysis since the culture had earlier reached maximum OD and went through a transient plateau phase. Prophage induced endolysis was evident only in the induction control of prophage harbouring-strain TMW 1.1290 treated with 5 µg/ml mitomycin C (Fig. 6).

### RT-qPCR of UV induced amidase gene

Following the exposure of UV light to prophage harbouring-strain TMW 1.1290, the relative expression ratio of the prophage gene amidase was analyzed using RT-qPCR. To normalize data, the geometric means of the Ct values for each time point were calculated using three housekeeping genes (*recA*, *gyrB* and *dnaG*). The determined efficiencies of the primers were considered sufficient for quantitative analysis (recA 98%, DNAgyr 91%, DNA primase 95%, Amidase 88%). The time points for sample collection were 1, 2 and 3 h after treatment and the calculation was compared to sample taken immediately after the treatment.

**Fig. 7:**
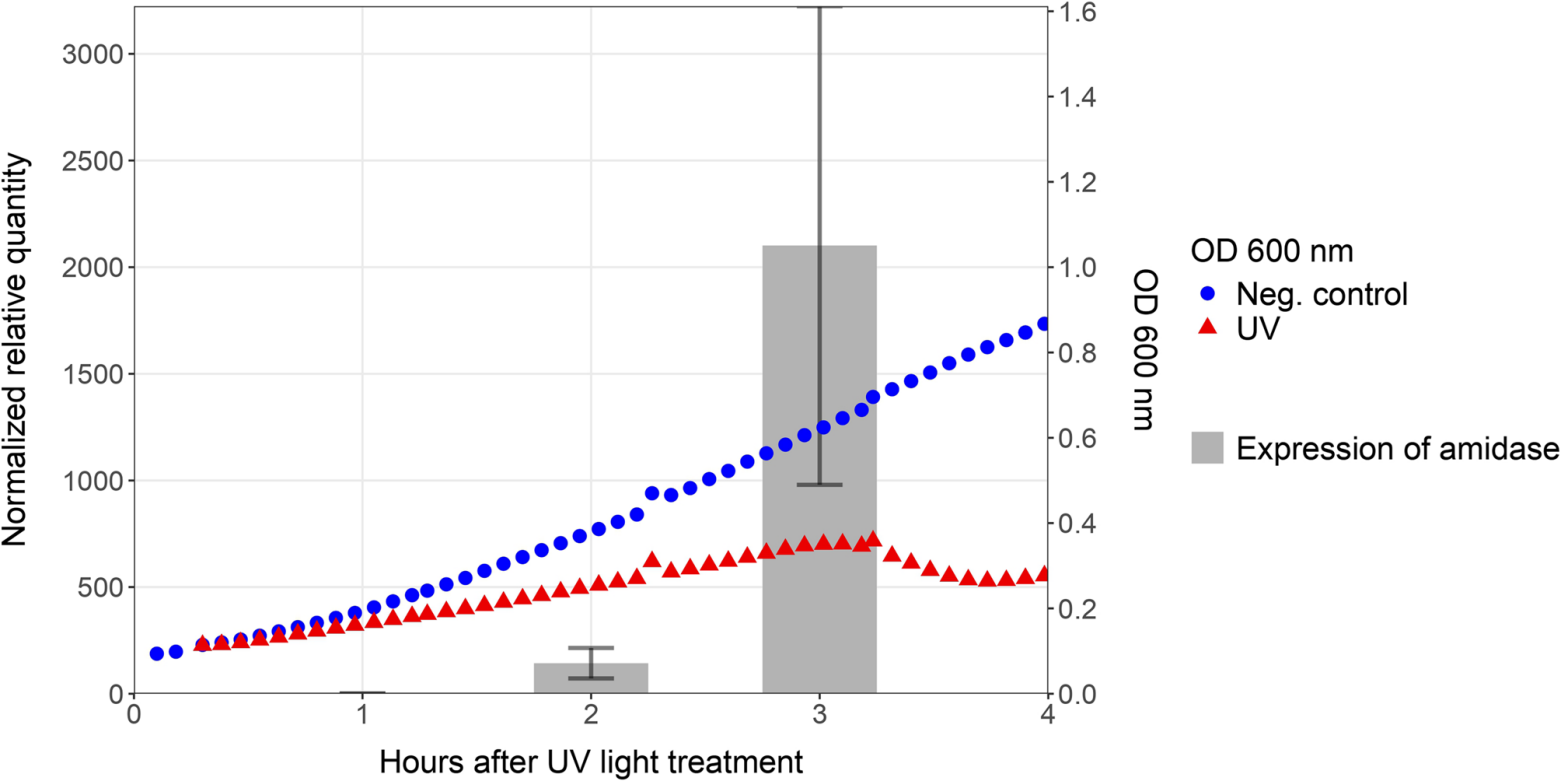
Relative expression ratio of the prophage amidase gene on different timepoints after the treatment with UV light *in vitro*. Ct values from RT-qPCR were compared to the control (0 h) and normalized to three housekeeping genes *recA*, *gyrB* and *dnaG*. The standard deviation (± 1 SD) is specified on the basis of two independent samples. The OD_600_ development shows the treated sample and untreated control

The amidase gene was found to be upregulated two hours after treatment, when it was approximately 143 times greater than directly after the treatment (Fig. 7). Three hours after the treatment, was the expression ratio approximately 2102 times greater. The values for the relative expression ratio of the amidase gene show a strong increase until the lysis of the cells began, which was observed with the decreasing absorbance. Further information about the raw data and Ct values are shown in the supplementary material.

## Discussion

Both growth conditions of treated cultures and intensity of treatment can have decisive effects on the development of OD_600_ and prophage induction, as already shown by [17]. Preliminary experiments defined the optimum timepoint of chemical induction at the early logarithmic phase. The dosage of inducer for each method, either chemical concentration or UV intensity, was selected at the threshold between causing mere growth inhibition and causing immediate cell death. Compared to earlier work of Ambros & Ehrmann [17] on the same strain, deviations in the effect of mitomycin C and UV light were seen. In our case, a slightly higher mitomycin C concentrations (5–20 µg/ml) were needed to trigger prophage induced lysis, which may be explained by a higher cell counts of the culture at the timepoint of mitomycin C addition. Furthermore, UV treatment did not result in induction of complete lysis, i.e. reduction of OD_600_ to near 0 as seen by Ambros & Ehrmann [17], but rather in a transient prophage induced lysis, followed by a second growth stage. This suggests that the irradiation effect was not homogeneous in the cell suspension or that the irradiation was obstructed. Although slightly different UVB light wavelengths were used in the two studies (302 vs. 312 nm), research has shown that within the wavelength range 300 to 315 nm, differences in DNA damaging effect are negligible [36]. In contrast, the penetration of UVB light, through borosilicate glass may be severely attenuated also depending on its thickness [37, 38] while the depth and turbidity of liquid can also have an effect on the germicidal activity of UVB [39, 40]. Taken together, failure to closely control these factors may introduce difficulties in reproducing the exact same effect of UVB irradiation on the induction of prophages and could explain the deviations seen in its effect between the two studies.

Treatment of prophage harbouring-strain TMW 1.1290 with mitomycin C resulted in a longer latency period and higher cell growth between treatment and start of cell lysis than any other treatment. Taking into account that cell counts, initial OD_600_ and growth stage of the cells were standardized across treatments, these differences may be explained by differences in the mode of action of the various inducers. DNA damage generally triggers the SOS response in bacteria which in turn induces the lytic replication cycle of phages leading to endolysis [16, 41]. In general, UVB light induces direct DNA damage by formation of thymine dimers and other DNA lesions which can be repaired. Mitomycin C on the other hand, selectively acts on the DNA, causing difficult to repair cross-linking of the DNA strands, leading to inhibition of DNA synthesis and eventual cell death [42]. While direct comparisons of the ability of different triggers, like UV and mitomycin C, to induce prophage lysis are rare in the literature, there is some evidence that mitomycin C is a more potent inducer than UV [43, 44]. Furthermore, in our experiments the damage caused by UVB treatment is confined to the duration of the treatment while mitomycin C may be present in sufficient concentration for an extended time during incubation and thus exert a cumulative damaging activity on the cells.

Agents like H_2_O_2_ and menadione have been shown to elicit prophage induced lysis in cells [44, 45] by generation of ROS, such as O_2_^−^, H_2_O_2_, and hydroxyl radicals (HO**·**) which can damage biomolecules in cells by altering the functionality of proteins and lipids or even causing DNA strand breaks [46]. In another study, a direct comparison between H_2_O_2_ and mitomycin C in their ability to induce lambdoid Shiga toxin-converting prophage ST2-8624 showed that induction with H_2_O_2_ was relatively rapid compared to that with mitomycin C [47]. This agrees with our results (Figs. 2 and 3), as OD_600_ reduction as observed earlier (~ 1 h after treatment) in H_2_O_2_ treated cell cultures than in mitomycin treated cultures (3–4 h after treatment).

In our study, differences were also observed in the effect of H_2_O_2_ and menadione on the prophage-free culture. Treatment of the control culture (TMW 1.2292) with menadione led to growth stagnation as evidenced by the unchanged OD_600_ of the culture for the whole incubation time (Fig. 4). This observation agrees with the results of Mone et al., [48] who showed that treatment of *Staphylococcus aureus* with menadione caused a lag phase in the culture while ROS gradually accumulated to lethal levels in the cells up to 9 h from treatment, after which point a rapid reduction in cell numbers occurred. Compared to menadione, the effect of H_2_O_2_ on the control culture in our study was transient growth retardation, followed by growth at seemingly the same growth rate as the not treated control culture Fig. 4.

Treatment with PEF, under the specific experimental procedure followed, failed to induce lysis in the prophage-harbouring strain TMW 1.1290 and had no effect on the growth of the control strain TMW 1.2292. Although, to our knowledge, there is no literature on the induction of prophages by use of PEF, application of PEF in aqueous cell-free solutions as well as in cell suspensions has been shown to generate superoxide anions (O_2_^•-^), hydrogen peroxide (H_2_O_2_), hydroxyl radicals (•OH) and singlet oxygen (^1^O_2_) [49], in the cytoplasm as well as in the extracellular environment. Pakhomova et al., [25] showed that ROS production in the cytoplasm of PEF treated Jurkat cells depended on field strength, frequency, and pulse number, with effects varying by cell type. Similarly, in yeast cells, Minamitani et al., [50] found that low-frequency PEF caused membrane oxidative damage, while higher frequencies led to mitochondrial damage and cytotoxicity.

The inability of PEF treatment to induce prophages, may be due to the failure of the treatment applied to cause an adequate concentration of intra- and/or extracellular ROS under the specific experimental procedure followed. Indeed, in our study the concentration of H_2_O_2_ required to elicit a prophage induction in prophage-harbouring strain TMW 1.1290 was calculated at a minimum of 2 mM. Comparatively, the concentration of hydrogen peroxide measured by Pakhomova et al. [25] in an aqueous cell suspension treated with 3000 pulses at 2 kV/cm, 300 ns and 30 Hz was a mere 1 µM which is approx. 2000 times lower than the concentration of H_2_O_2_ applied in our experiments in which induction of prophage was observed. Although the settings of our PEF treatment deviated considerably from and cannot be directly compared with those applied by Pakhomova et al., [25], the comparison suggests that a much more intense PEF treatment would have to be applied in our experiments in order to achieve a concentration of intra- and/or extracellular ROS high enough to cause prophage induction. Notably, a small but statistically significant reduction of the cell counts of prophage-harbouring strain TMW 1.1290 was already observed after PEF treatment with the highest specific energy in our experiments (PEF 2), suggesting that an even more severe treatment would be counterproductive in causing adequate prophage induction as it would cause further reduction in cell counts.

Treatment of the prophage harboring strain with HHP failed to elicit prophage induced lysis. Early research on the effect of HHP on microorganisms has shown that it can cause the creation of fibrillar regions of DNA in the cytoplasm, associated with the effect of endonucleases [51, 52]. Furthermore, more recent research has shown the induction of DNA damage related SOS response genes in *Listeria monocytogenes* within 10 min after HHP treatment at 4000 bar [28]. Nevertheless, HHP is not considered to cause breakage of the double-stranded DNA [53] except maybe indirectly, due to generation of ROS in the cells [54]. Although evidence that HHP can cause the induction of prophages is rare in literature, there is published evidence of this in *L. monocytogenes* [55] as well as with the induction of lambda prophages and lambdoid Stx-converting bacteriophages in *Escherichia coli* MG1655 [21, 22]. In the latter, this was associated with the increased expression of LexA and RecA proteins, initially suggesting an SOS response to DNA lesions caused by the treatment [21]. Later studies, however, on the same *E. coli* strain showed that HHP treatment caused the dissociation of the tetramer Mrr endonuclease to its active dimer. This altered the specificity of the enzyme and led to cleavage of the cell’s own DNA [56, 57] which could explain the induction of LexA and RecA proteins. A similar effect of HHP on endonucleases in lactobacilli, however, has not been reported. With all this in mind and considering that the intensity of HHP treatment used in our experiments was enough to cause a small but significant reduction in cell counts of prophage harbouring-strain TMW 1.1290, it is unclear why HHP failed to cause prophage induction.

## Conclusions

This study demonstrated that prophage-mediated cell lysis in prophage harbouring-strain *L. sakei* TMW 1.1290 can be induced by specific treatments, particularly UV light and mitomycin C. Although oxidative stress through H_2_O_2_ and menadione showed potential for prophage activation, it resulted in significant growth inhibition. Food technologies, potentially causing oxidative stress, like PEF and HHP were ineffective, under the specific conditions used, despite reaching intensities that caused sublethal injury. Further investigation is needed to determine the effect of PEF and HHP treatment settings on the ability of the treatments to induce prophage mediated cell-lysis.

## Data Availability

Availability of data and materials: The genome of prophage harbouring-strain *Latilactobacillus Sakei* TMW 1.1290 can be accessed under accession number JAHIAJ000000000 at the NCBI website.
